# Single-Sensor Engine Multi-Type Fault Detection

**DOI:** 10.3390/s23031642

**Published:** 2023-02-02

**Authors:** Daijie Tang, Fengrong Bi, Jiangang Cheng, Xiao Yang, Pengfei Shen, Xiaoyang Bi

**Affiliations:** 1 State Key Laboratory of Engines, Tianjin University, Tianjin 300350, China; 2 State Key Laboratory of Reliability and Intelligence Electrical Equipment, Hebei University of Technology, Tianjin 300130, China

**Keywords:** fault detection, single-sensor data, variational mode decomposition, vibration, random forest

## Abstract

Engine fault detection is conducive to improving equipment reliability and reducing maintenance costs. In practical scenarios, high-quality data is difficult to obtain. Usually, only single-sensor data is available. This paper proposes a fault detection method combining Variational Mode Decomposition (VMD) and Random Forest (RF). At first, the spectral energy distribution is obtained by decomposing and statistic the engine data of multiple working conditions. Based on the spectral energy distribution, the overall optimal mode number was identified, and the quadratic penalty term was optimized using SNR. The improved VMD (IVMD) improves mode aliasing and iterative efficiency and unifies feature dimensions. Decomposition of real signals demonstrates the effectiveness. The paper designs a feature vector composed of seven types of attributes, including unit bandwidth energy, center frequency, maximum singular value and so on. The feature vector is then fed to RF for classification. Features are selected in order of importance to classification to improve the training efficiency. By comparing with various algorithms, the proposed method has higher accuracy and faster training efficiency in single-speed, multi-speed and cross-speed single-sensor data diagnosis. The results show that the method has application prospects with little training data and low hardware requirements.

## 1. Introduction

As one of the critical power sources, the reliability of engines has received more attention in recent years. In time, engine fault detection can detect weak faults, which is conducive to fault prevention and repair. Data-driven approaches usually require large amounts of high-quality data for training. However, engine labelled-data is challenging to obtain and mostly comes from a single sensor due to cost constraints. Research on single-sensor engine fault detection based on small data amounts and low hardware requirements is necessary [1].

Vibration acceleration signals are widely used in fault detection research because of their rich component condition information and ease of measurement [2,3]. Ma et al. proposed a multi-channel Lanczos quaternion singular spectrum analysis to extract fault characteristic frequencies from multiple vibration sensor signals [4]. Ribeiro et al. proposed a multi-head one-dimensional convolutional neural network to diagnose six motor faults using vibration signals from two directions [5]. However, the engine vibration signal has a wide frequency band (up to about 12,000 Hz), and sensors with high sampling accuracy and a wide frequency band with good stability are usually costly. Moreover, the hardware conditions of the engine control system are ordinary, so the research on single-sensor fault detection under low hardware requirements is gradually gaining attention. Basuraj et al. proposed a single-sensor online filtering method for recursive singular spectrum analysis based on the concept of first-order feature perturbation, which proved its effectiveness in several data sets. [6]. Ayati et al. used KNN for single-sensor fault classification after extracting features using fast Fourier transform and wavelet packet transform [7]. In general, it is challenging to diagnose faults in different cylinders of an engine separately using a single sensor.

Engine fault detection methods can be roughly divided into three categories: knowledge-driven, model-driven, and data-driven. Wang et al. proposed an aero-engine dynamic threshold fault detection based on the isolated forest method, which requires only normal data for training to achieve high accuracy [8]. Ellefsen et al. proposed an online diagnosis method for marine diesel engine degradation based on variational autoencoder and expert knowledge [9]. Knowledge-driven methods usually diagnose a single type of fault and require solid expert knowledge. Liu et al. proposed a model-based aero-engine soft fault detection method, which achieved fault diagnosis by comparing smooth residuals and preset thresholds [10]. Wang et al. established a mapping model between the shaft radial vibration average and the misalignment value based on shaft shape characteristics. A new monitoring scheme has been designed and the accuracy of detecting misalignment is greater than 90% [11]. Model-driven method research can help explore the failure mechanism, but it is usually challenging to achieve. Data-driven methods are widely used due to their ease of implementation and high accuracy [12,13]. Deep learning methods have been widely used in engine fault detection in recent years due to their powerful data mining capabilities [14,15]. These methods require less expert knowledge and more high-quality training data. However, high-dimensional and huge data processing capability leads to higher hardware requirements for deep learning methods. In practical scenarios, high-quality training data is difficult to obtain because of the dangers of engine failure simulation experiments. Under the constraints of low hardware conditions and lack of data, the combination of signal processing methods and simple pattern classification methods still has potential to be explored [16,17].

Variational Mode Decomposition (VMD) is an advanced signal processing method capable of decomposing a signal into several intrinsic mode functions (IMFs) [18]. Compared with empirical mode decomposition (EMD), VMD effectively suppresses mode aliasing and improves the quality of decomposition [19]. However, the mode number K and quadratic penalty term α, predefined in VMD, strongly influence the decomposition and are difficult to determine [20,21]. For these reasons, scholars have proposed many optimization ideas for adaptively selecting K and α [21,22]. The adaptive VMD method leads to a varying number of IMFs, so component screening is usually performed after decomposition [23,24]. The process of screening IMFs requires expert knowledge and is time-consuming and labor-intensive. In addition, many scholars optimize (K, α) through swarm intelligence optimization algorithms [25,26]. This method ignores the problem that the VMD efficiency drops sharply as K increases (as shown in Section 4).

The unsupervised clustering method is ineffective in diagnosing engine faults because there are many types of failure, complex operating conditions, and large signal noise [27,28]. Supervised pattern classification methods such as deep neural networks (DNN) are more suitable due to their powerful learning capabilities. Shahid et al. used a one-dimensional convolutional neural network (1DCNN) to identify the crankshaft angle degree of the engine and successfully diagnosed the misfire fault [29]. Zhang et al. proposed a long short-term memory recurrent neural network (LSTM-RNN) for evaluating bearing degradation and proposed waveform entropy to improve the accuracy effectively [30]. Lee et al. compared the performance of multilayer perception (MLP), residual network (ResNet), LSTM, and ResNet-LSTM in diagnosing production failure cases and found that ResNet-LSTM works best [31]. However, the effectiveness of DNN is built on sufficient high-quality labeled data. Due to the complex calculation of DNN, the training time is long, and it is challenging to optimize and retrain the model [32]. Li et al. first used a simplified DNN to extract the fault features of rotating machinery and then combined random forest (RF) for fault classification, which has higher efficiency and accuracy than advanced DNN methods [32]. RF has faster training and classification speed than DNN and may be suitable for engine fault detection.

The paper aims to propose a single-sensor, cross-speed fault detection method that is applicable to low hardware requirements and small data amounts. The work has resulted in the following contributions.

(1) A new overall K and α optimization method based on spectral energy distribution and SNR is proposed for VMD, avoiding IMF screening and unifying the feature dimension to prepare for quick diagnosis.
(2) The center frequencies are preset based on spectral energy distribution, which reduces the number of VMD iterations and mode aliasing.
(3) A feature set was designed for IVMD-RF to achieve single-sensor fault diagnosis. Further filtering of features by feature importance ranking improves efficiency. Different single-sensor datasets demonstrate the effectiveness of the method.

The rest of the article is organized as follows. Section 2 introduces the basic principles of the methods used in the paper. In Section 3, the fault data collection experiment of the diesel engine is presented. Section 4 introduces the optimization of the VMD method and the verification of its decomposition effect. In Section 5, IVMD-RF is presented and compared with various DNN methods on two diagnostic cases.

## 2. Theories

### 2.1. Variational Mode Decomposition

The purpose of VMD is to decompose an actual signal into several ideal narrowband signals while satisfying the constraint that the sum of their bandwidths is the smallest. Assume that each IMF closely surrounds its center frequency in the frequency domain. Therefore, the objective can be summarized as the following constrained variational problem:

(1) $$\left\{ \begin{array}{c} \underset{\left\{u_{k}\right\},\left\{\omega_{k}\right\}}{\min}\{{\sum_{k}\left\|\partial_{t}[(\delta (t)+\frac{j}{\pi t})*u_{k}(t)]e^{-j\omega_{k}t}\right\|}_{2}^{2}\}\\ s.t.\sum_{k}u_{k}=f \end{array},\right.$$

where $\left\{u_{k}(t)\}=\{u_{1}(t),u_{1}(t),\ldots ,u_{k}(t)\right\}$ and $\left\{\omega_{k}\}=\{\omega_{1},\omega_{2},\ldots ,\omega_{k}\right\}$ represent the decomposed IMFs and the corresponding center frequencies, respectively. δ(t) is the shock function.

The reconstruction constraint can be addressed by introducing a quadratic penalty α and Lagrange multipliers λ. The constrained variational problem of (1) is transformed into an unconstrained one by introducing these two parameters. The obtained augmented Lagrangian is shown in (2):

(2) $$L\left(\left\{u_{k}\right\},\left\{\omega_{k}\right\},\lambda \right):=\alpha \sum_{k}{\left\|\partial_{t}[(\delta (t)+\frac{j}{\pi t})*u_{k}(t)]e^{-j\omega_{k}t}\right\|}_{2}^{2}+{\left\|f(t)-\sum_{k}u_{k}(t)\right\|}_{2}^{2}+\langle \lambda (t),f(t)-\sum_{k}u_{k}(t)\rangle ,$$

This problem can be solved by Parseval/Plancherel Fourier isometry under the norm. The expressions of ${\overset{⌢}{u}}_{k}^{n+1}(\omega )$ and $\omega_{k}^{n+1}$ are shown in (3) and (4).

(3) $${\overset{⌢}{u}}_{k}^{n+1}(\omega )=\frac{\overset{⌢}{f}(\omega )-\sum_{i\neq k}{\overset{⌢}{u}}_{i}(\omega )+\frac{\overset{⌢}{\lambda }(\omega )}{2}}{1+2\alpha {\left(\omega -\omega_{k}\right)}^{2}},$$

(4) $$\omega_{k}^{n+1}=\frac{\int_{0}^{\infty }\omega {\left|{\overset{⌢}{u}}_{k}(\omega )\right|}^{2}d\omega }{\int_{0}^{\infty }{\left|{\overset{⌢}{u}}_{k}(\omega )\right|}^{2}d\omega },$$

where (3) is equivalent to the Wiener filter of the current residual $\overset{⏜}{f}\left(\omega \right)-\sum_{i<K}\overset{⏜}{u_{i}}\left(\omega \right)$. IMF can be obtained by inverse Fourier transform of ${\overset{⌢}{u}}_{k}^{n+1}(\omega )$.The flow of VMD is shown in Algorithm 1. The default ε value is 1 × 10^−7^.
**Algorithm 1**: VMD**Input**: A signal f, mode number K and quadratic penalty α. **Output**: A set of IMFs Initialize $\left\{{\overset{⏜}{u}}_{k}^{1}\},\{{\overset{⏜}{\omega }}_{k}^{1}\},\{{\overset{⏜}{\lambda }}^{1}\right\}$, n ← 0 **repeat** **for** k ← 1 to K **do**
Update ${\overset{⌢}{u}}_{k}$ for all ω≥0 by (3) Update $\omega_{k}$ by (4) **end for** Dual ascent for all ω≥0:  ${\overset{⏜}{\lambda }}^{n+1}(\omega )\leftarrow {\overset{⏜}{\lambda }}^{n}(\omega )+\tau \left[\overset{⏜}{f}(\omega )-\sum_{k}{\overset{⏜}{u}}_{k}^{n+1}(\omega )\right]$ **until convergence**: $\sum_{k}{\left\|{\overset{⏜}{u}}_{k}^{n+1}-{\overset{⏜}{u}}_{k}^{n}\right\|}_{2}^{2}/{\left\|{\overset{⏜}{u}}_{k}^{n}\right\|}_{2}^{2}<\varepsilon$.

### 2.2. Random Forests

The random forest algorithm was proposed by Breiman [33], which is suitable for solving data prediction and classification. A random forest is a combination of decision tree classifiers. Each tree depends on the value of an independently sampled random vector and has the same distribution for all trees in the forest.

(1) Suppose the original sample is $X=\{(x_{1},y_{1}),(x_{2},y_{2}),\ldots ,(x_{n},y_{n})\}$, where $x_{i}$ and $y_{i}$ represent feature values and labels, respectively. T training samples $X_{1},X_{2},\ldots ,X_{T}$ are extracted from the original dataset *X* by bootstrap sampling with return, and $X_{i}(i=1,2,\ldots T)$ and *X* have the same number of samples.
(2) Build a decision tree $hi(X_{i},\Theta_{k})$ for each training sample $X_{i}(i=1,2,\ldots T)$, where i =1, 2, …T, k =1, 2, …. The decision tree model used in the paper is shown in (5) and (6).

(5) $$d(x_{1},x_{2},\ldots ,x_{n},h_{t})=\left\{\begin{array}{l} label(h_{t})\;\;\;h_{t}\;\mathrm{is}\;\mathrm{the}\;\mathrm{leaf}\;\mathrm{node}\\ d(x_{1},x_{2},\ldots ,x_{n},h_{t})\;h_{t}\;\mathrm{is}\;\mathrm{the}\;\mathrm{inner}\;\mathrm{node}\; \end{array}\right.$$

(6) $$hi(X_{i},\Theta_{k})=d(x_{1},x_{2},\ldots ,x_{n},root(h_{t}))$$

where *root*(*h_t_*) is the root node of the decision tree. $d(x_{1},x_{2},\ldots ,x_{n},h_{t})$ is the division criterion of the decision tree. The segmentation criterion consists of segmentation variables and predictions measured by the impurity function.

The Gini coefficient is proportional to the impurity level. The optimal split is to find the largest split of the Gini coefficient as follows:

(7) $$Gini(t)=1-\sum_{j=1}^{J}{\left\{p(j|t)\right\}}^{2}$$

where p(j|t) is the probability of the *j*th category in node *t*, that is, the ratio of the *j*th category to the total number of sample labels *J*.

Before selecting attributes for each non-leaf node, randomly select *m* attributes from *M* attributes as the set of categorical attributes for the current node. Take $m=\mathrm{int}(\sqrt{M})$, where int is the rounding function. The nodes are divided according to the optimal division method of *m* attributes, and a complete decision tree is established. The growth of each decision tree is not pruned until the leaf node grows.

A random forest generated from *T* decision trees is used to classify the test samples. Each tree has voting power to decide the classification result. Summarize the output categories of the decision tree, and the category with the most votes is the final classification result. The classification decision model *H(x)* is shown in (8).

(8) $$H(x)=\arg\underset{\gamma }{\max}\sum_{i=1}^{T}I(hi(X_{i},\Theta_{k})=\gamma )$$

where *γ* is the label variable of the output and I is the indicator function.

## 3. Diesel Engine Faults Simulation Experiment

**Figure 1:**
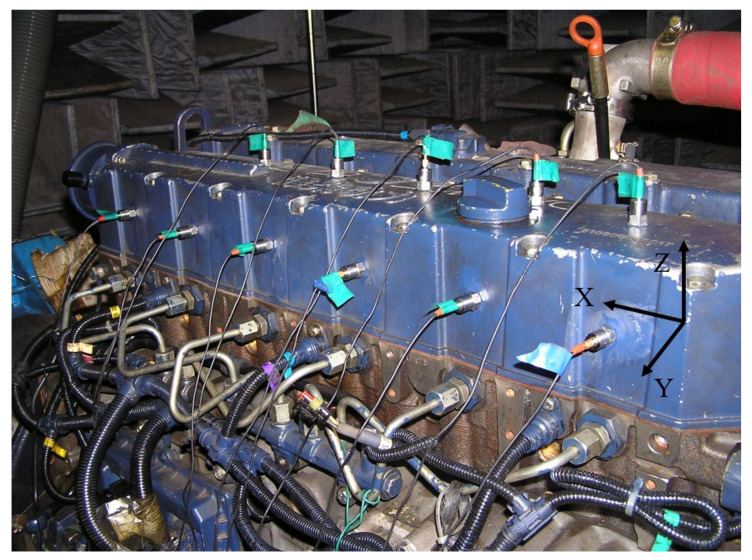
Sensor positions and coordinate direction.

To verify the effectiveness of the proposed method, our team conducted a fault simulation experiment on an in-line 6-cylinder diesel engine. The specific parameters of the engine are shown in Table 1. The experiment was performed on a bench base supported by an air spring. The engine and the dynamic dynamometer adopt a flexible connection. The photoelectric pulse speed sensor is placed at the position of the vertical connecting shaft to measure the engine speed. The vibration acceleration sensors are arranged on the cylinder head and block as shown in Figure 1. The data used in this paper are vibration acceleration signals in the Y-direction in Figure 1. The signal is input to the computer for processing and recording after passing through the acquisition front end. The models of the instruments used in the experiment are shown in Table 2.

Components with the highest failure probability are fuel injection and oil supply equipment (25.1%), water leakage (13.1%), and valves and sealing (17.4%) [34]. Generally, leak failure is easy to detect by water temperature sensors. The paper focuses on two other types of failures. The paper simulates three faults for the fuel supply equipment: abnormal common rail pressure, abnormal fuel supply, and abnormal injection advance angle. Abnormal rail pressure is set to simulate the fault of the common rail system, and the insufficient fuel supply is to simulate injector failure. The weak power combustion abnormality is simulated by slightly changing the injection advance angle. In addition, the abnormal valve clearance is simulated by adjusting the opening of intake and exhaust valves with a plug gauge. The abnormal valve clearance conditions all occurred on the first cylinder only. Experiments were performed at the following rotational speeds: 700 rpm, 1300 rpm, 1600 rpm, 2000 rpm, and 2300 rpm. The parameters of normal working conditions under each speed condition are shown in Table 3. The fault settings at rated speed (2300 rpm) are shown in Table 4, where the Roman numerals represent different fault conditions. The fault conditions of other speeds are also adjusted to the same extent as those in Table 4 on the basis of the normal parameters in Table 3. The abnormal advance angle failure simulation is not carried out under 700 rpm idling conditions. The load range of the engine includes 100% and 50%.

## 4. Optimization of Variational Mode Decomposition

**Figure 2:**
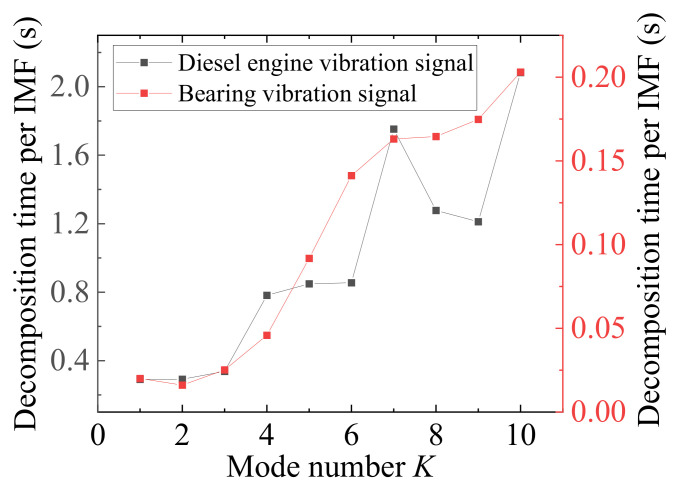
Decomposition time per IMF of VMD. The curve data come from the average of normal signals of various speeds. The engine data come from the experiment of Section 3. The bearing data come from the bearing dataset of Case Western Reserve University [37].

VMD’s denoising ability is better than EMD [35], and the decomposed IMFs have a better signal-to-noise ratio (SNR). However, the decomposition effect of VMD is greatly affected by parameter settings, especially the mode number *K* and the quadratic penalty term *α*. Improper *K* value setting will lead to over-decomposition or under-decomposition. In addition, as *K* increases, the efficiency of the original VMD decreases drastically. Figure 2 shows the effect of different *K* values on the decomposition time of each IMF. The results show that the efficiency of VMD is much higher when *K* ≤ 3. From Figure 2, traversing *K* to find the optimal value and using various swarm intelligence optimization algorithms are both inefficient. Therefore, Ref. [36] proposes an adaptive recursive variational mode decomposition (ARVMD) that dynamically selects the *K* in recursive loops. ARVMD effectively improves efficiency and reduces recursive mode aliasing. The process of ARVMD is shown in Algorithm 2.
**Algorithm 2**: ARVMD **Input**: A signal *f*_0,_ Sampling frequency Fs and quadratic penalty *α*. **Output**: A set of IMFs. *f* = *f*_0_; IMFs = []; E_u_ = []; *i* = 0; **while** E_ui_ > E_th_ do *i* = *i* + 1; P*_f_*←Power spectral density (*f*); (P_max_, F_max_) ←Maximum, corresponding frequency (P*_f_*); N_peak_ ←Numbers of maxima points in [F_max_ ± 0.027 × Fs]; {F_1_, F_2_, …, F_n_}← Corresponding frequencies of maxima points; $K_{i}=\left\{\begin{array}{c} 1,N_{peak}<2\\ 2,N_{peak}=2\\ 3,N_{peak}>2 \end{array}\right.$ {*u*_1_, *u*_2_,…, *u*_Ki_} ← VMD (*f*, *K_i_*, *α*, {F_1_, F_2_, …, F_Ki_}); {E_u1_, E_u2_, …, E_uKi_}← Unit bandwidth energy ({*u*_1_, *u*_2_,…, *u*_Ki_}); IMFs ←IMFs ∪{*u*_1_, *u*_2_,…, *u*_Ki_}; E_u_ ←E_u_ ∪{E_u1_, E_u2_, …, E_uKi_}; *f* = *f*
$-\sum_{1}^{Ki}u_{Ki}(t)$; **end while** IMFs ←Selection by E_ui_ > E_th_ (IMFs) **return** IMFs 

**Figure 3:**
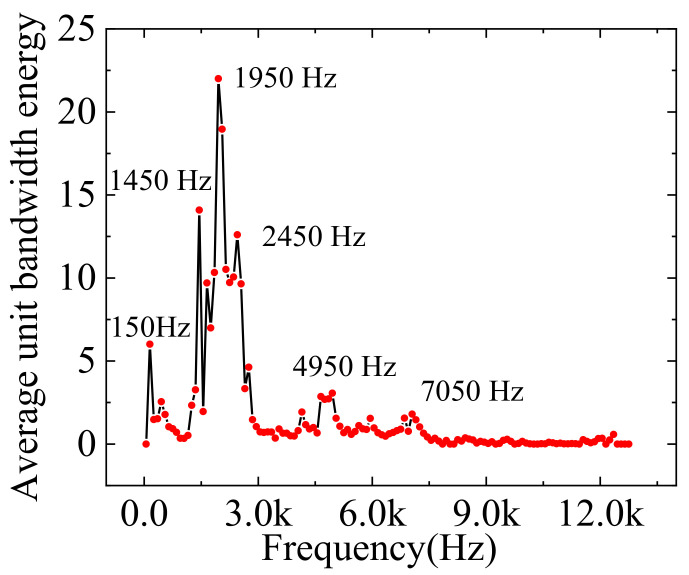
Frequency domain distribution of unit bandwidth energy of engine data.

Complex types and working conditions characterize engine faults. However, the component number obtained by ARVMD is variable, resulting in inconsistent feature vector dimensions, which is not conducive to diagnosing multi-speed engine vibration data. A *K*-value optimization method based on the energy distribution in the frequency domain is proposed to unify the feature dimension. First, ARVMD decomposes the signals of various engine working conditions and obtains many IMFs. These conditions contain data for different speeds and faults (I to XII, as shown in Table 4). Then, the unit bandwidth energy [36] of each IMFs is calculated, and the center frequency of the IMFs is recorded. The unit bandwidth energy is shown in (9):

(9) $$E_{u}=\frac{e_{IMFi}}{B_{IMFi}}$$

where $e_{IMFi}$ is the energy of IMF and $B_{IMFi}$ is the bandwidth of IMF. The bandwidth is the width of the spectrum when the amplitude of the power spectral density is reduced by 99%. The frequency band [0 Hz, 12,800 Hz] is divided into 128 segments, and the width of each segment is 100 Hz. The unit bandwidth energy of the components located in each segment is counted and averaged. Figure 3 shows the unit bandwidth energy spectrum displayed on divided frequency bands. The results show that there are six prominent energy frequencies: 150 Hz, 1450 Hz, 1950 Hz, 2450 Hz, 4950 Hz, and 7050 Hz. Here, each frequency segment uses the frequency in the middle as the value of the abscissa. Therefore, the engine data will be uniformly decomposed using *K* equal to 6. This approach can improve the consistency of data processing and help reduce the randomness caused by adaptive decomposition. It also ensures that the dimension of the feature vectors at different speeds is uniform.

Furthermore, the iterations of the center frequency of the original VMD are zero-based. It is beneficial for decomposing low-frequency components, but the decomposition time for high-frequency components is longer. Presetting suitable initial center frequencies can significantly improve the efficiency of the VMD [18]. Therefore, the six significant frequencies in Figure 3 are used as the initial center frequencies to iterate.

**Figure 4:**
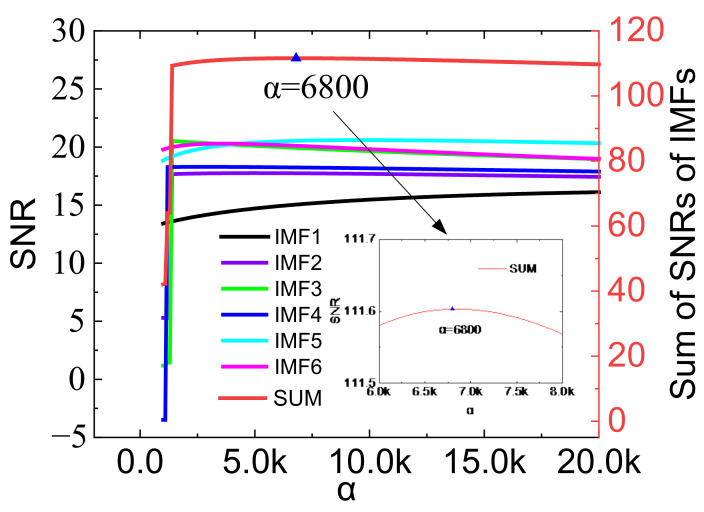
The effect of α on the decomposition SNR.

The quadratic penalty term *α* is a parameter introduced to improve the convergence when solving the variational model. The role of α in the decomposition is reflected in the noise reduction of the signal. The SNR is the best criterion for choosing a suitable α. However, it is difficult to obtain the SNR of the actual signal after decomposition. Therefore, a set of simulated signals is constructed according to the spectral energy distribution of Figure 3. The expression of the simulated signal is as (10). {*s_1_*, *s_2_*, …, *s_6_*} are single-frequency components, which restore the amplitude ratio and frequency of each component in Figure 3. The amplitude of *s_3_* is set to 100, and the other components are reduced proportionally. *s_7_* is the noise component with a power of 25 dbW. *S_1_* is decomposed using VMD, where *K* is six, and the initial center frequency is preset. Set the variation range of α to [1000, 20,000], and the step size is 100. Calculate the SNR between IMFs and {*s_1_*, *s_2_*, …, *s_6_*}, and the results are shown in Figure 4. With the increase of *α*, the SNR has a trend of increasing first and then decreasing. Summing the SNR of each component, it is found that the total SNR does not change much when α is 6000 to 8000. The value of α used in the paper is 6800, and the inset of Figure 4 shows that the SNR reaches the maximum at this value. After the optimized α is obtained, it is used in the mode number optimization for reverse verification, and the results show that it does not affect the results in Figure 3.

(10) $$\left\{\begin{array}{l} s_{1}\left(t\right)=27\sin\left(2\pi *150t\right),0\leq t\leq 0.053\\ s_{2}\left(t\right)=64\sin\left(2\pi *1450t\right),0\leq t\leq 0.053\\ s_{3}\left(t\right)=100\sin\left(2\pi *1950t\right),0\leq t\leq 0.053\\ s_{4}\left(t\right)=57\sin\left(2\pi *2450t\right),0\leq t\leq 0.053\\ s_{5}(t)=13\sin\left(2\pi *4950t\right),0\leq t\leq 0.053\\ s_{6}(t)=8\sin\left(2\pi *7050t\right),0\leq t\leq 0.053\\ s_{7}(t)=\eta \\ S_{1}=s_{1}+s_{2}+s_{3}+s_{4}+s_{5}+s_{6}{+s}_{7} \end{array}\right.$$

**Figure 5:**
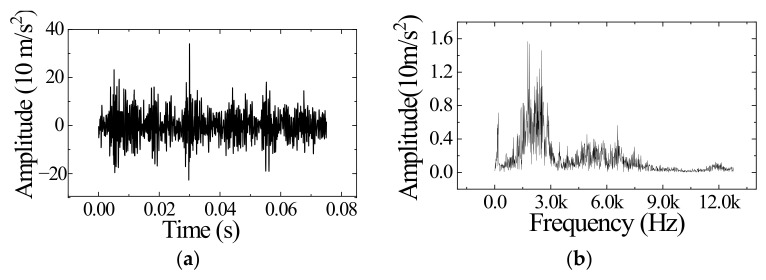
A signal of valve clearance increase at 1600 rpm. (**a**) The signal in time domain. (**b**) The signal in frequency domain.

**Figure 6:**
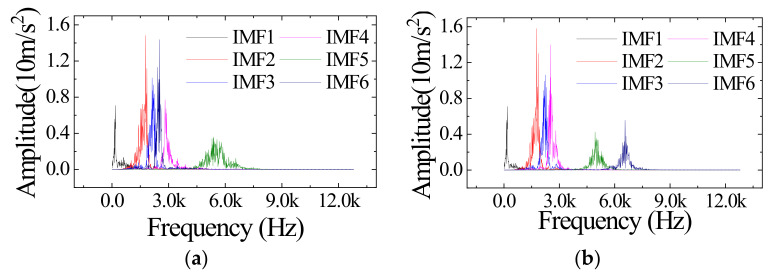
Frequency domain image of the decomposed IMFs. (**a**) Result of VMD. (**b**) Result of IVMD.

**Figure 7:**
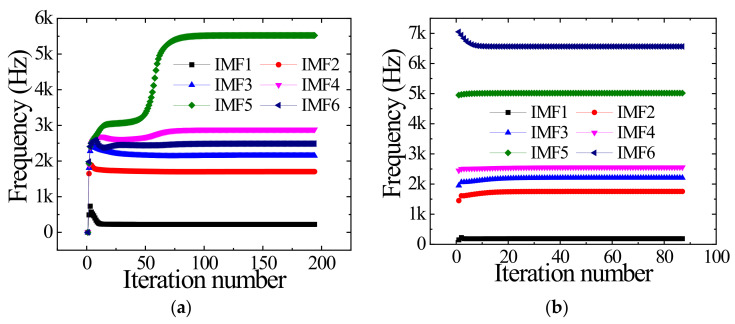
Center frequency iterative curves for IMFs. (**a**) Iterative curves of VMD. (**b**) Iterative curves of IVMD.

The optimization of *K*, *α* and the iterative optimization of the center frequency have been completed. Next, decompose an actual signal using the improved VMD (IVMD) to verify the effect. A signal of valve clearance increase at 1600 rpm (Condition III in Table 4) was randomly selected for decomposition. The signal’s time and frequency domain are shown in Figure 5a,b. Decompose this signal using VMD and IVMD. Figure 6 shows the frequency domain image of the decomposed IMFs. VMD decomposes four components in the [2000 Hz, 3000 Hz] while IVMD decomposes three. The results show that using the same *K*, VMD focuses on decomposing low-frequency components, while IVMD is more balanced. The average bandwidth aliasing ratio *R_ABA_* is introduced to measure the effect of suppressing mode aliasing [36]. The expression of *R_ABA_* is shown in (11):

(11) $$R_{ABA}=\sum_{i=1}^{K}\frac{1}{K}\frac{B_{A}}{B_{IMFi}},i=1,2,\ldots ,K.$$

where *K* is the mode number, *B_A_* is the aliasing bandwidth of the IMFi and other components, and B_IMFi_ is the bandwidth of IMFi. The smaller the *R_ABA_*, the better the effect of suppressing mode aliasing. The *R_ABA_* for VMD and IVMD results is 0.13 and 0.05, respectively. IVMD suppresses mode aliasing better than VMD. In addition, the center frequency iteration curves of IMFs are shown in Figure 7. Figure 7 shows that IVMD performs 87 iterations, less than VMD’s 194 iterations, effectively improving efficiency. The results show that the presetting center frequency can significantly improve the iteration efficiency.

## 5. VMD-RF Fault Detection Method

After the IVMD decomposition of the engine signal, calculating proper features is beneficial to improve the diagnostic accuracy. The RF method can automatically select a subset of features for classification by bootstrap sampling with return. Instead of considering the feature dimension, features are required to describe the data information as comprehensively as possible. Therefore, the used features include overall features and local features. Finally, seven types of local features are selected. Namely, maximum singular value, energy, unit bandwidth energy, kurtosis, variance, root mean square value (RMS), and center frequency. The seven types of features are calculated for the six IMFs obtained by IVMD. In addition, maximum singular value, energy, RMS, and variance are calculated for the original signal. Feature names and symbols are shown in Table 5.

### 5.1. Case 1: Diesel Engine Fault Diagnosis

**Figure 8:**
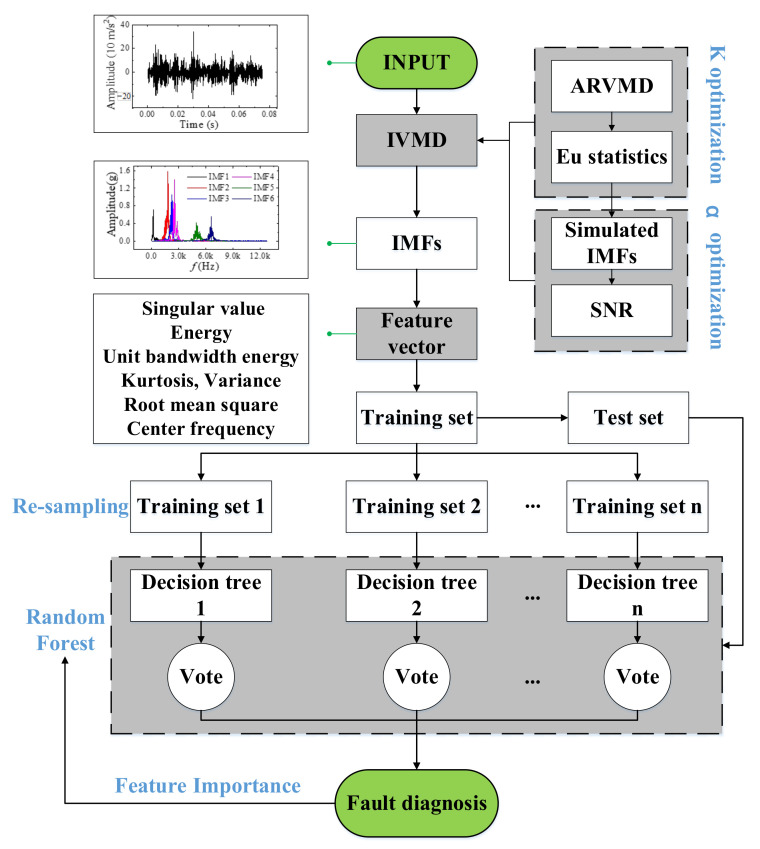
IVMD-RF fault detection process.

Once the complete feature set is obtained, the feature set can be fed into the RF for classification. The whole flow of fault diagnosis is shown in Figure 8. The data of the first cylinder head (1H), the third cylinder head (3H), and the first cylinder block (1B) at 2300 rpm and the data of the first cylinder head at 2000 rpm were selected for preliminary verification of the algorithm’s validity. Each dataset contains four types of faults in Table 4, with a total of 12 fault conditions. Each fault condition includes 200 samples, and the training/test ratio is 4:1. The number of decision trees in RF is 100. The depth of the decision tree is not limited. Then, the training samples are used to generate a random forest. The results of the diagnostic accuracy are shown in Table 6. The proposed method is compared with sequential minimum optimization for support vector machines (SMO-SVM), Multilayer Perceptron (MLP) [31], one-dimensional convolutional neural networks (1DCNN) [29], long and short term memory recurrent neural networks (LSTM-RNN) [30], and residual neural networks (ResNet) [31]. The 1DCNN and LSTM-RNN ran for 200 epochs, while ResNet ran for 30 epochs. The parameters of each algorithm are as follows:

(1) SVM: The RBF kernel is chosen, and the penalty term C is set to 1. The inverse of the radius of influence of the support vector gamma is set to 0.1.
(2) MLP: Two hidden layers are used, both with 30 neurons. The momentum is 0.2, and the learning rate is 0.3.
(3) 1DCNN: The network consists of two convolutional layers (kernel size = 5), two maximum pooling layers (kernel size = 2), and a linear layer. The activation function is ReLU, and the optimizer is Adam.
(4) LSTM-RNN: The network contains two LSTM layers with 64 nodes in each layer.
(5) ResNet: The network uses the 18-layer ResNet model, as described in Ref. [38].

**Table 6:** Accuracy comparison of different algorithms (%).

Methods	1H-2000 rpm	1H-2300 rpm	1B-2300 rpm	3H-2300 rpm	1H-Multi-Speed
SMO-SVM	99.06	97.50	93.75	92.91	92.59
MLP	97.81	98.13	92.36	92.71	96.79
1DCNN	94.06	99.06	92.89	94.06	96.56
LSTM-RNN	59.06	75.16	78.12	68.75	56.23
ResNet	91.09	96.88	88.28	86.72	92.19
IVMD-RF	98.75	99.38	92.91	93.54	97.32

Note: “3H” represents the third cylinder head, “1B” represents the first cylinder block.

SMO-SVM, MLP, 1DCNN, and IVMD-RF achieved high accuracy from the results of single-speed data. The LSTM-RNN had the lowest accuracy, which shows its poor classification ability for non-time series. Compared to the 1H data set, the diagnostic accuracy of the 1B and 3H datasets decreased significantly due to the increased distance of the sensor location from the combustion chamber and valve. The vibration signal may be distorted or coupled with other disturbances when it is transmitted.

The next step is to use these methods to diagnose multiple speed conditions. All types of failure data for the first cylinder head (1H) at 700 rpm, 1300 rpm, 1600 rpm, 2000 rpm, and 2300 rpm were made into one dataset. Since there are no abnormal injection advance angle faults in the 700 rpm data, a total of 56 labeled categories of data are included. The diagnostic results are shown in Table 6. Compared to the single-speed dataset for the first cylinder head (1H), the accuracy of each method decreases to varying degrees as the number of failure types increases. The accuracy of SMO-SVM dropped the most. SMO-SVM method is suitable for single-speed data classification but not as effective as other methods for multi-speed and multi-class data. The proposed method still maintains high accuracy. The results show that IVMD-RF has advantages for multi-speed and multi-type fault diagnosis scenarios.

In addition, the proposed method requires less training time to achieve high accuracy. For comparison, all algorithms are run in the same environment (Python 3.8, Windows 11, Intel Core i7-10700 CPU @ 2.9 GHz), and the running time is recorded in Table 7 The results show that SMO-SVM has the highest training efficiency, followed closely by IVMD-RF. ResNet has the longest training time due to the deep network layers. Therefore, the proposed method has high efficiency and high accuracy. It is worth noting that deep learning may provide better diagnostic results for the original raw signal. However, the significant increase in data dimensionality leads to an increase in computation time and higher hardware requirements, which deviates from the purpose of this paper. No diagnostics were performed on the original raw data to keep the variables consistent.

**Figure 9:**
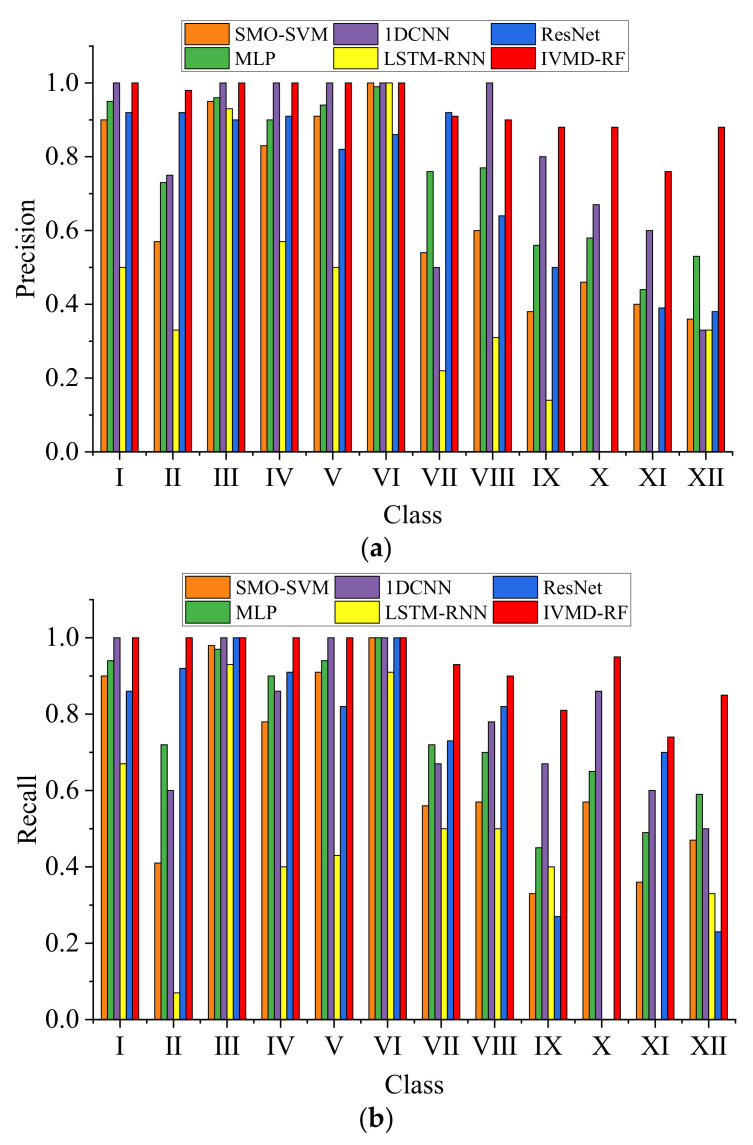
Precision and recall results of each algorithm for the 3H dataset. (**a**) Precision result. (**b**) Recall result.

**Figure 10:**
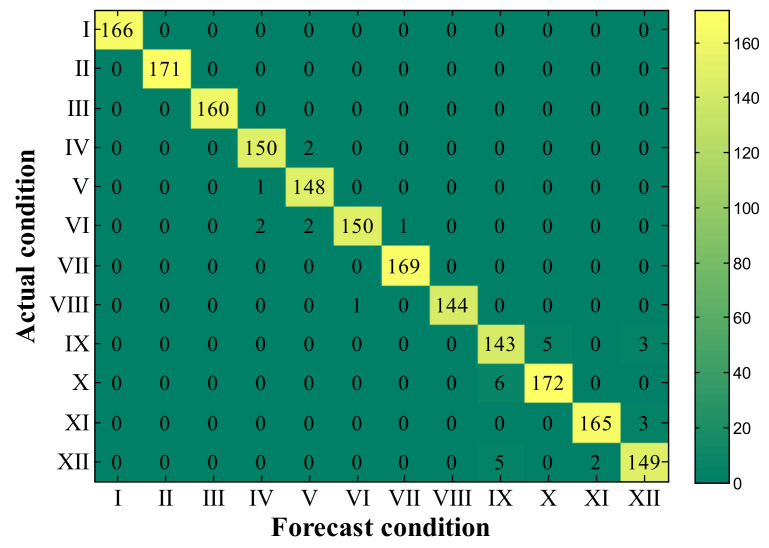
Confusion matrix for 1H dataset of IVMD-RF.

Next, the 1300 rpm, 1600 rpm, 2000 rpm, and 2300 rpm data were mixed into one dataset. The data were labeled into 12 categories according to Table 4, regardless of the speed change. The cross-speed datasets include the 1H dataset, 1B dataset, and 3H dataset. The above methods are still used for classification, and the results are shown in Table 8. The results show that the accuracy of each algorithm has a certain drop compared to Table 6, especially the SMO-SVM. The 1DCNN and IVMD-RF still maintain a relatively high accuracy rate. The overall accuracy of the 3H dataset is low because the sensor in the third cylinder head is far from the cylinder where some failures occurred. The proposed method still has some advantages over other algorithms. Table 8 also shows the classification precision, recall, and f1-score. These indicators are weighted averages, where the weights are determined by the proportion of each class sample distribution. The results show that the proposed method also performs well on these metrics. For the 3H dataset with relatively poor diagnostic results, Figure 9 shows the comparison of recall and precision of each algorithm for the 12 classes. The results showed low recall and accuracy for reduced valve clearance (condition II) and abnormal injection advance angles (condition IX to XII). The reason for the low precision and recall of Fault II is the slight increase in valve clearance and the long distance of the sensor from the cylinder where the fault occurred. Faults IX to XII, on the other hand, are due to small changes in injection advance angle, causing only minor differences in combustion conditions. The training efficiency of the proposed method is much higher than that of the deep learning method and slightly lower than that of SMO-SVM. Figure 10 shows the confusion matrix for the 1H dataset, indicating that most of the misclassified samples are data of the same type but with different failure levels, which proves the effectiveness of the proposed method.

**Figure 11:**
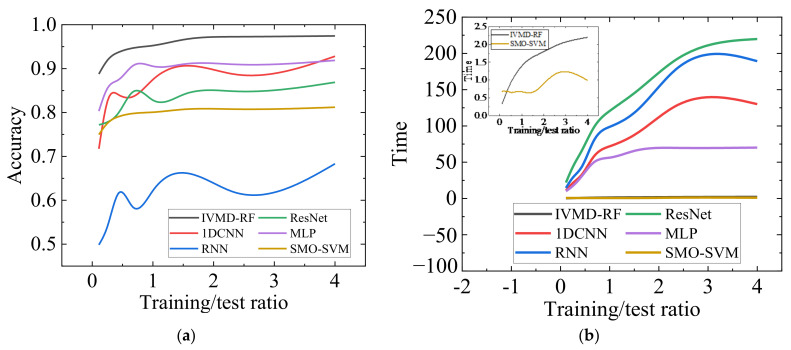
Comparison of each algorithm with different training/testing ratios (0.1 to 4). (**a**) Comparison of accuracy. (**b**) Comparison of the training time.

**Figure 12:**
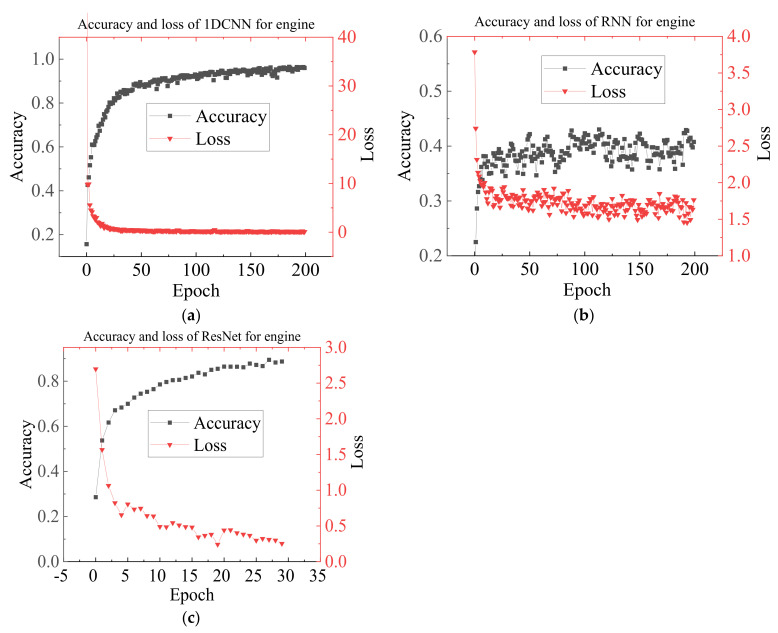
Accuracy and training loss of deep learning methods for 1H dataset. (**a**) Accuracy and training loss of 1DCNN. (**b**) Accuracy and training loss of LSTM-RNN. (**c**) Accuracy and training loss of ResNet.

The datasets with different training/testing ratios are set up for classification to verify the diagnostic effectiveness of various methods for the small sample case. Figure 11 shows each algorithm’s accuracy and time consumption curves for the 1H dataset at different training test ratios (0.1 to 4). When the training test ratio <0.25, the accuracy of 1DCNN, RNN, and MLP significantly decrease, while SMO-SVM and IVMD-RF decrease more smoothly. When the training test ratio is 0.1, IVMD-RF has the highest accuracy of 88.77%. Figure 11b shows that the training efficiency of each algorithm increases as the training/test ratio decreases. The efficiency of IVMD-RF and SMO-SVM remains higher than the other methods. The results illustrate the good diagnostic effect of the proposed method for small samples of single-sensor data. Figure 12 shows the accuracy and training loss curves when the three deep learning methods are applied to the 1H dataset. Figure 12 indicates that the 1DCNN has converged while the RNN clearly shows over-fitting, which is the reason for its low accuracy. Continuing to train ResNet may improve the accuracy, but the training efficiency is too low compared to other methods. Therefore, IVMD-RF has a high fault diagnosis accuracy and high efficiency for cross-speed data. It is worth noting that deep learning methods still have more advantages and potential when the amount of labeled data and computational resources are sufficient.

**Figure 13:**
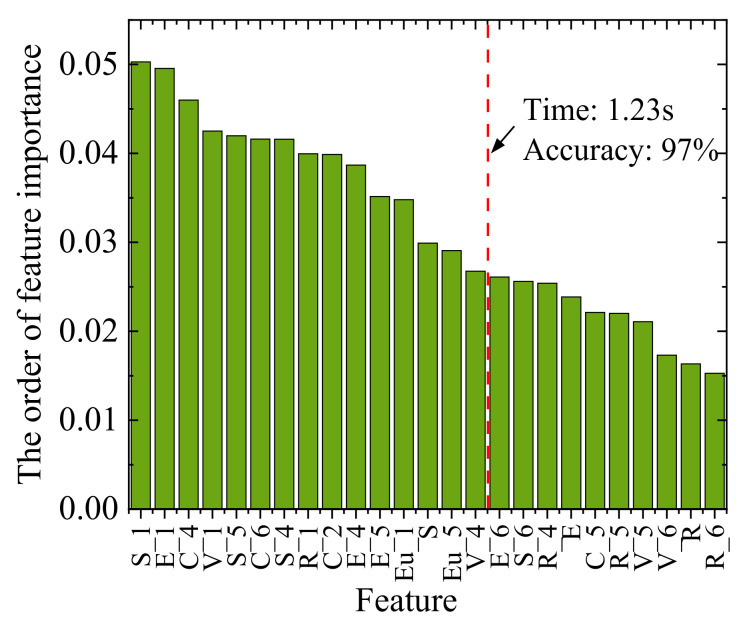
The order of feature importance.

**Figure 14:**
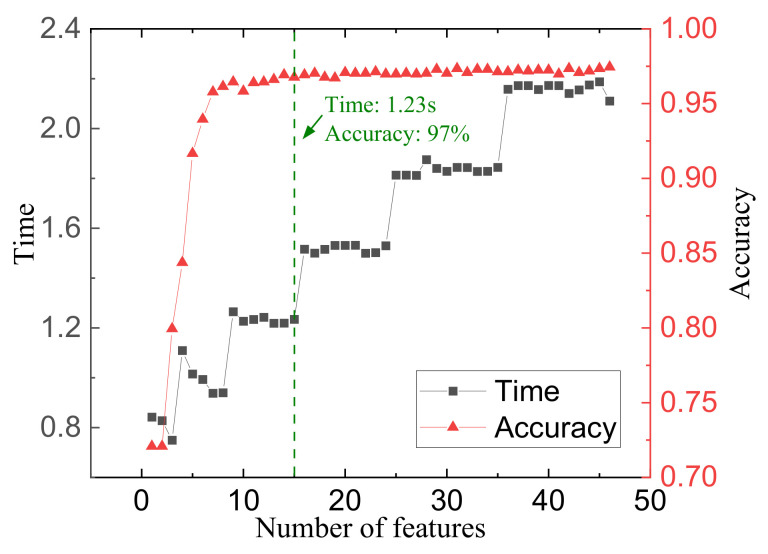
The impact of changing the number of features used.

The study of feature importance can further improve the performance of the method. Using the RF to rank the importance of features, the results for the 1H dataset are shown in Figure 13. Singular values, energy, and center frequency contributed more to the classification, followed by variance and unit bandwidth energy. However, kurtosis has little contribution to the classification results. Figure 13 shows that features with suffixes 1 and 4 contribute significantly to the classification, i.e., IMF1 and IMF4 contribute the most to the classification, followed by IMF5 and IMF6. For different working conditions, the difference in the body surface vibration is mainly reflected in the low-frequency (IMF1) and high-frequency components (IMF4~6). IMF2 and IMF3 have high energy but weak contribution. This conclusion is valuable for the study of unsupervised engine fault diagnosis. Figure 14 shows the impact of using different numbers of features in order of importance on training time and accuracy. Finally, we found an optimal point. When using fifteen features, it only takes 1.23 s to train and can achieve 97% diagnostic accuracy as marked in Figure 14. The selected fifteen categories of features are marked in Figure 13. Feature selection significantly improves training time with little change in accuracy.

### 5.2. Case 2: Gasoline Engine Fault Diagnosis

**Figure 15:**
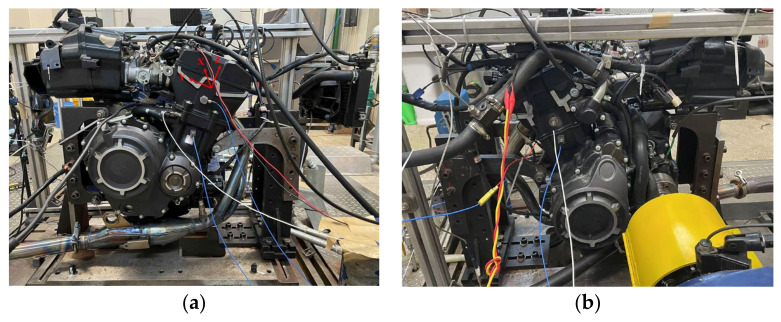
Sensor positions and coordinate direction of the engine. (**a**) Position of sensor 1Y. (**b**) Position of sensor 2X.

To verify the effectiveness of the proposed method on different engines, the gasoline engine fault data will be diagnosed in the following. The fault data came from a two-cylinder, two-stroke gasoline engine with the specific engine parameters shown in Table 9. The sensor locations and coordinate system for the engine are shown in Figure 15. The data used are from the Y-direction of cylinder 1 and the X-direction of cylinder 2 (the two sensors connected by the white wire in Figure 15). Three common faults were simulated: abnormal injection advance angle, abnormal air-fuel ratio, and misfire. Among them, the abnormal injection advance angle occurs in both cylinders, while the other two failures occur only in cylinder 1. The specific fault level settings are shown in Table 10. Use Roman numerals I through VII to indicate individual faults. Signals from 5000 rpm and 7000 rpm were collected. Each working condition contains 200 samples, each containing data from one working cycle.

The 5000 and 7000 rpm data were mixed to form the cross-speed dataset. Various algorithms diagnose the fault data of 1Y and 2X sensors separately. The settings of each algorithm are shown in Section 5.1. A comparison of the diagnostic results for each algorithm is shown in Table 11. The results show an overall decrease in the diagnostic accuracy of each algorithm due to the increase in signal noise as the two-cylinder, two-stroke engine vibrates more than the diesel engine. The higher speed is also one of the reasons. SMO-SVM is still the fastest, but its accuracy is low. The proposed method works best for fault diagnosis of 1Y data, and 1DCNN works best for 2X data. The difference in accuracy between the two is not significant. The proposed method is more efficient and suitable for low hardware conditions. The results show that IVMD-RF can be used for gasoline engine fault diagnosis.

## 6. Conclusions and Discussion

This article proposes an IVMD-RF for single-sensor multi-fault detection of the engine. In IVMD, the engine data spectral energy distribution is obtained through multiple decompositions and statistics. The alpha value was chosen based on the spectral distribution and the SNR. By presetting the center frequency and the optimal *K* and α values, the efficiency is improved, the mode aliasing is reduced, and the feature size is unified. The effectiveness of IVMD is proved by decomposing the engine signals. Seven types of attributes are calculated to form a feature group for IMFs, which is input into RF for classification. Compared with various machine learning and deep learning algorithms, it is proved that the proposed method has advantages in training efficiency and accuracy. Through the feature importance study, it is found that the high-frequency and low-frequency IMFs contribute more to the classification. Fifteen optimal features have been selected to improve the efficiency of RF. The IVMD-RF method has application prospects in engine single-sensor multi-class fault detection.

## Figures and Tables

**Table 1:** Parameters of diesel engine.

Items	Parameters
Displacement	7.14 L
Rated power/Rated speed	220 kW/2300 rpm
Maximum torque/Speed range	1250 Nm/1200–1600 rpm
Intake/Exhaust valve clearance	0.30 m/0.50 m

**Table 2:** Experimental instrument parameters.

Instruments	Parameters
Dynamic dynamometer	CAC380, Xiangyi Power
Vibration acceleration sensor	621B40, PCB
Photoelectric pulse speed sensor	SPSR-115/230, Monarch
Data acquisition front end	SCADAS05, LMS

**Table 3:** Normal working conditions under different speeds.

Speed (rpm)	Valve Clearance-Intake, Exhaust (mm)	Fuel Supply (mg/cyc)	Rail Pressure (bar)	Injection Advance Angle (°CA)
700	(0.30, 0.50)	60.0	405	-
1300	(0.30, 0.50)	117.0	1250	9.49
1600	(0.30, 0.50)	117.0	1350	12.98
2000	(0.30, 0.50)	117.0	1500	15.00
2300	(0.30, 0.50)	112.5	1550	18.45

**Table 4:** Fault type and degree parameter setting (2300 rpm).

Mark	Valve Clearance (Intake, Exhaust)/mm	Fuel Supply	Rail Pressure/bar	Injection Advance Angle/°CA
I	(0.30, 0.50)	100%	1550	18.45
II	(0.20, 0.40)	100%	1550	18.45
III	(0.35, 0.55)	100%	1550	18.45
IV	(0.40, 0.60)	100%	1550	18.45
V	(0.30, 0.50)	75%	1550	18.45
VI	(0.30, 0.50)	25%	1550	18.45
VII	(0.30, 0.50)	100%	1350	18.45
VIII	(0.30, 0.50)	100%	1150	18.45
IX	(0.30, 0.50)	100%	1550	17.45
X	(0.30, 0.50)	100%	1550	16.45
XI	(0.30, 0.50)	100%	1550	19.45
XII	(0.30, 0.50)	100%	1550	20.45

Note: The shaded green marks the location of the faulty parameter.

**Table 5:** Attribute names and symbols.

Attribute Name	Symbols
Maximum singular value	S_1, S_2, S_3, S_4, S_5, S_6
Energy	E_1, E_2, E_3, E_4, E_5, E_6
Unit bandwidth energy	Eu_1, Eu_2, Eu_3, Eu_4, Eu_5, Eu_6
Kurtosis	K_1, K_2, K_3, K_4, K_5, K_6
Variance	V_1, V_2, V_3, V_4, V_5, V_6
Root mean square	R_1, R_2, R_3, R_4, R_5, R_6
Center frequency	C_1, C_2, C_3, C_4, C_5, C_6
Original signal attributes	S, E, R, V

Note: The features calculated for IMF1 to IMF6 are denoted by the symbols with suffixes 1 to 6, respectively. The symbols without suffixes indicate the features calculated for the original signal.

**Table 7:** Comparison of training time of various algorithms (s).

Methods	1H-2000 rpm	1H-2300 rpm	1B-2300 rpm	3H-2300 rpm	1H-Multi-Speed
SMO-SVM	0.05	0.06	0.05	0.07	2.84
MLP	9.60	9.91	9.55	9.53	274.21
1DCNN	13.02	14.10	14.65	14.87	167.38
LSTM-RNN	17.85	18.21	20.26	20.48	224.95
ResNet	49.32	47.66	54.53	51.66	446.92
IVMD-RF	0.26	0.28	0.31	0.29	4.30

Note: “3H” represents the third cylinder head, “1B” represents the first cylinder block.

**Table 8:** Comparison of fault diagnosis results of each algorithm for cross-speed dataset.

/	Methods	SMO-SVM	MLP	1DCNN	LSTM-RNN	ResNet	IVMD-RF
First cylinder head Y-direction (1H)	Accuracy	0.81	0.92	0.93	0.68	0.87	0.97
	Precision	0.82	0.92	0.94	0.65	0.89	0.97
	Recall	0.81	0.92	0.92	0.63	0.88	0.96
	F1-score	0.82	0.92	0.92	0.62	0.88	0.97
	Time (s)	0.99	70.19	130.02	189.29	219.77	2.20
First cylinder block Y-direction (1B)	Accuracy	0.81	0.89	0.93	0.67	0.86	0.92
	Precision	0.81	0.89	0.94	0.75	0.87	0.92
	Recall	0.80	0.89	0.91	0.72	0.85	0.92
	F1-score	0.80	0.89	0.91	0.72	0.86	0.92
	Time (s)	1.13	70.69	145.99	192.59	224.21	2.74
Third cylinder head Y-direction (3H)	Accuracy	0.65	0.75	0.83	0.47	0.78	0.94
	Precision	0.66	0.76	0.83	0.42	0.72	0.93
	Recall	0.65	0.76	0.80	0.42	0.71	0.94
	F1-score	0.65	0.76	0.81	0.40	0.71	0.93
	Time (s)	1.03	70.28	143.31	203.47	221.34	2.92

**Table 9:** Parameters of gasoline engine.

Items	Parameters
Displacement	0.294 L
Rated power/Rated speed	35.5 kW/8500 rpm
Maximum torque/Speed range	44.5 Nm/7000 rpm

**Table 10:** Fault type and degree parameter setting.

Mark	Injection Advance Angle	Air/Fuel Ratio	Misfire Rate
I	10 °CA	1	0
II	5 °CA	1	0
III	15 °CA	1	0
IV	10 °CA	1.1	0
V	10 °CA	1.2	0
VI	10 °CA	1	0.05
VII	10 °CA	1	0.1

Note: The shaded green marks the location of the faulty parameter.

**Table 11:** Comparison of fault diagnosis results of each algorithm for gasoline engine data.

	Methods	SMO-SVM	MLP	1DCNN	LSTM-RNN	ResNet	IVMD-RF
First cylinder head Y-direction (1Y)	Accuracy	0.67	0.75	0.82	0.52	0.78	0.85
	Precision	0.68	0.77	0.81	0.69	0.78	0.85
	Recall	0.67	0.75	0.82	0.51	0.79	0.84
	F1-score	0.67	0.72	0.82	0.52	0.78	0.84
	Time (s)	0.33	15.83	9.45	6.44	11.02	1.16
Second cylinder head X-direction (2X)	Accuracy	0.72	0.74	0.82	0.59	0.79	0.79
	Precision	0.72	0.74	0.82	0.58	0.77	0.78
	Recall	0.72	0.74	0.81	0.58	0.76	0.79
	F1-score	0.72	0.74	0.82	0.58	0.74	0.79
	Time (s)	0.25	15.80	9.61	6.51	11.05	1.12

## Data Availability

The data presented in this study are not publicly available at this time but may be obtained upon reasonable request from the authors.
